# Histological Heterogeneity of Primary Liver Cancers: Clinical Relevance, Diagnostic Pitfalls and the Pathologist’s Role

**DOI:** 10.3390/cancers13122871

**Published:** 2021-06-08

**Authors:** Mina Komuta

**Affiliations:** 1 Department of Pathology, International University of Health and Welfare, School of Medicine, Chiba 286-8520, Japan; mina-komuta@iuhw.ac.jp or mina.komuta@gmail.com; 2 Department of Pathology, School of Medicine, Keio University, Tokyo 160-8582, Japan; 3 Department of Pathology, School of Medicine, Kurume University, Fukuoka 830-0011, Japan

**Keywords:** hepatocellular carcinoma, cholangiocarcinoma, combined hepatocellular-cholangiocarcinoma, cholangiolocellular carcinoma, keratin 19 positive hepatocellular carcinoma, molecular profiles, pathological diagnosis, tumor heterogeneity, liver cancer, differential diagnosis

## Abstract

**Simple Summary:**

Primary liver cancers (PLCs) mainly comprise hepatocellular carcinoma (HCC), intrahepatic cholangiocarcinoma (iCCA), and combined (c)HCC-CCA. Both small duct types iCCA (a subtype pf iCCA) and cHCC-CCA are known to be tumors with histological heterogeneity. Understanding key tumor heterogeneity is crucial as it reflects tumor aggressiveness, patient outcome, treatment choice, and is predictive of treatment efficacy. In addition, PLCs often present with multiple liver tumors, which can be a combination of different types of PLCs or HCCs (intrahepatic metastasis or multicentric occurrence), and the pathological interpretation plays an important role in these cases. The aim of this review is to clarify the pathological features of HCC, iCCA, and cHCC-CCA, including their diagnostic pitfalls, molecular profiles, and the correlation between tumor subtypes and treatment choice.

**Abstract:**

Primary liver cancers (PLCs) mainly comprise hepatocellular carcinoma (HCC), intrahepatic cholangiocarcinoma (iCCA), and cHCC-CCA. Combined HCC-CCA and small duct type iCCA show similar clinical presentations, and their histological features are more complex than seen in HCC. Therefore, while their treatment strategy differs, it is difficult to properly diagnose these tumors. Currently, HCC is the only tumor that can be treated by liver transplantation. In addition, small duct type iCCA harbors IDH1/2 mutations and FGFR2 fusions, which can be used for targeted therapy. Thus, improving diagnostic accuracy is crucial. A further point to note is that PLCs often present as multiple liver tumors, and they can be a combination of different types of PLCs or HCCs. In the case of HCCs, two different scenarios are possible, namely intrahepatic metastasis, or multicentric occurrence. Therefore, it is essential to characterize the type of multiple liver tumors. This review aims to clarify the pathological features of HCC, iCCA and cHCC-CCA, including their diagnostic pitfalls and clinical relevance. It is designed to be of use to clinicians who are dealing with PLCs, to provide a better understanding of the pathology of these tumors, and to enable a more accurate diagnosis and optimal treatment choice.

## 1. Introduction

**Figure 1:**
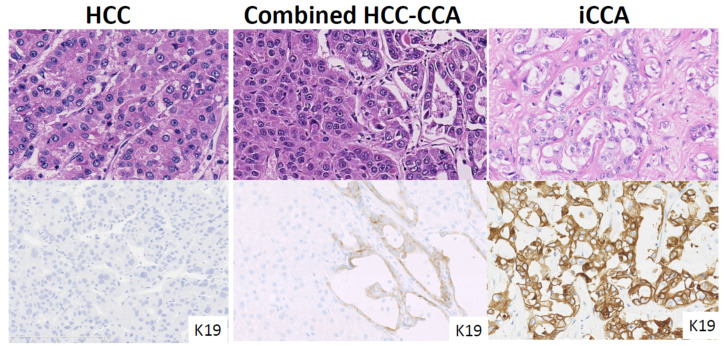
Pathological features of hepatocellular carcinoma (HCC), intrahepatic cholangiocarcinoma (iCCA) large duct type, and combined HCC-CCA. HCC shows a trabecular growth pattern, whereas large duct type iCCA shows a glandular structure with mucin production. Combined HCC-CCA shows both HCC and iCCA features. Keratin 19 staining shows diffuse and intense cytoplasmic positivity in iCCA, but negative in HCC. Combined HCC-CCA shows K19 weak expression in the cholangiocytic differentiated area.

Primary liver cancers (PLCs) mainly comprise hepatocellular carcinoma (HCC), intrahepatic cholangiocarcinoma (iCCA), and cHCC-CCA (Figure 1). As indicated by their names, HCC stems from hepatocytes, and iCCA originates from cholangiocytes lining the epithelia of the biliary tracts. Despite the simplicity of the names given to PLCs, their pathological diagnosis is not always straightforward, especially in the case of iCCA and cHCC-CCA, with their complex pathological features, described as “histological heterogeneity” [1,2]. For instance, cHCC-CCA comprises both hepatocytic and cholangiocytic differentiation. The percentage of both components varies depending on the tumor, and as such, the images and clinical presentations may vary as well. For instance, if the hepatocytic component is predominant, the tumor will present with more HCC-like features in terms of images and clinical features, and vice versa.

In the case of iCCA, the pathological features are similarly complex. iCCA comprises two subtypes, namely small duct type and large duct type, according to the 5th WHO classification [3], and they are totally different. Firstly, the mass-forming type is commonly seen in small duct type iCCA, but not in large duct type iCCA. Secondly, small duct type iCCA is associated with underlying liver diseases and the tumor may present with some vascularity, similar to HCC. Therefore, small duct iCCA needs to be distinguished from both HCC and cHCC-CCA.

The treatment strategy differs depending on the tumor. For instance, liver transplantation is currently only used for HCC, and not for cHCC-CCA or iCCA. Additionally, recent studies show that small-duct type iCCA harbors IDH1/2 mutations, or FGFR2 fusions, which can be utilized for targeted therapy. In fact, clinical trials of both treatments show promising data [4,5,6].

Thus, it is essential to diagnose PLCs correctly as it affects the treatment decision. Due to their histological complexity and similar clinical presentations, pathological interpretation is extremely useful for diagnosis.

This review aims to clarify the pathological features of HCC, iCCA and cHCC-CCC, including diagnostic pitfalls and clinical relevance, thus enabling a better understanding of these tumors for more accurate diagnoses, and better treatment choices for patients.

## 2. Hepatocellular Carcinoma (HCC)

### 2.1. Histological Features

Hepatocellular carcinoma originates from mature hepatocytes, which are the main components of the liver. It is, therefore, understandable that HCC is the most frequent PLC, with an incidence around 75–85% of all PLCs [7].

Normal liver parenchyma appears as a trabecular pattern composed of mature hepatocytes with abundant eosinophilic cytoplasm, round nuclei, and distinct nucleoli. Mucin production is not seen in the hepatocytes. The HCC structure mimics this normal parenchyma, exhibiting a trabecular growth pattern composed of eosinophilic tumor cells without mucin production. As the tumor becomes less differentiated, the tumor cells are more basophilic, and tumor structures take on a thick trabecular/solid structure. The 5^th^ WHO classification defines eight HCC subtypes; steatohepatitic, clear cell, macrotrabecular-massive, scirrhous, chromophobe, fibrolamellar carcinoma, neutrophil-rich, and lymphocyte-rich [7].

HCCs are known to be hyper vascular tumors [8], as the tumor comprises abundant vascular structures, such as sinusoidal capillarization and unpaired arteries [9]. Blood stroma in HCC mimic the sinusoidal space in normal liver parenchyma; known as sinusoidal capillarization. HCC also contains unpaired small arteries, i.e., without biliary ducts and/or portal veins. HCCs are often encapsulated by a fibrous capsule. The HCC is fed by hepatic arteries that originate from the tumoral center and extend through to the tumoral periphery and out from the HCC through the fibrous capsule. Microvascular invasions are therefore often seen within, and/or at the periphery of the fibrous capsule. These are identified as microvascular invasions, and if they grow, they are recognized as satellite lesions.

### 2.2. Tumor Differentiation

Tumor differentiation reflects the aggressiveness of HCCs. As with other tumors, the tumor differentiation of HCCs comprises three categories; well, moderate, and poorly differentiated. Well differentiated HCC shows a thin trabecular growth pattern, mimicking a normal liver architecture. In contrast, moderately differentiated HCC shows a thick trabecular growth pattern (>four liver plates). Nodule in nodule appearance, one of the unique features of HCC, shows a well differentiated HCC in the periphery, and less tumor differentiation inside the tumor [10]. Most importantly, there is a big difference between well differentiated HCCs and moderate, and/or poorly differentiated HCCs, in terms of tumor aggressiveness. Microvascular invasion, and/or intrahepatic metastasis, and/or satellite lesions, are common in moderate and/or poorly differentiated HCC, however, they are hardly seen in well differentiated HCC [11].

### 2.3. Important HCC Pathology Reflecting Tumor Aggressiveness

#### 2.3.1. Macro Trabecular-Massive HCC

The macro trabecular-massive subtype is a poorly differentiated HCC that accounts for 12% of HCCs [12]. Morphologically, the tumor comprises thick trabeculae (>6 cells in thickness) coated by endothelial cells and surrounded by vascular spaces. It is defined when a macrotrabecular growth pattern is observed in >50% of the tumor. It often associates with vascular invasion and satellite lesions. Clinical outcome is poor due to early and overall recurrence. Importantly, this pattern can be identified in both surgical specimens and biopsied samples. It is essential to note this on the diagnostic protocol.

#### 2.3.2. Keratin 19 Positive HCC and a Diagnostic Pitfall When Distinguishing from cHCC-CCA and iCCA

**Figure 2:**
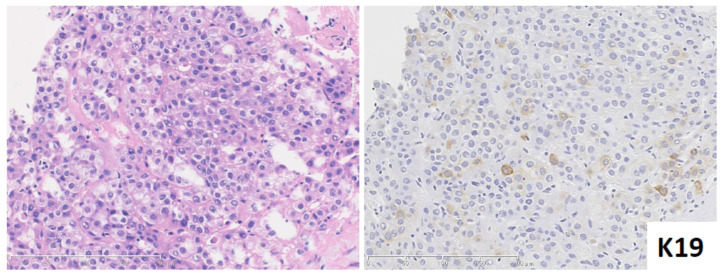
Keratin (K) 19 positive HCC. HCC shows moderate differentiation with no sign of glandular feature. K19 immunohistochemistry shows weak membranous and/or cytoplasmic positivity in the tumoral cells. (**Left**) hematoxylin and eosin staining; (**Right**) K19 immunohistochemistry.

Keratin 19 positive HCC presents with a typical HCC morphology, without glandular or mucin production (Figure 2). However, K19 immunohistochemistry reveals a weak cytoplasmic and/or membranous positivity of >5% of the HCC tumoral cells. K19 positive HCC is typically more aggressive, with earlier recurrence and shorter survival rates, compared with K19 negative HCC [13]. In addition, molecular profiles also confirm distinct invasive properties of K19 positive HCC [14]. HE staining cannot differentiate this subtype and it is therefore important to identify potential K19 positive HCCs using K19 immunohistochemistry.

It is also important not to misdiagnose K19 HCC as cHCC-CCA or iCCA [2]. Firstly, K19 positive HCC shows no sign of glandular structures and/or cholangiocarcinoma component [13]. Secondly, K19 positive HCC never presents with mucin production. Pseudo glandular structure, a common feature of HCC, may mimic the glandular structures seen in adenocarcinoma, however the absence or presence of mucin can be useful to distinguish between them. K19 immunoreactivity is also different between K19 positive HCC and cHCC-CCA or iCCA. K19 positive HCC usually shows very focal K19 expression, and the staining pattern is always weak and membranous. In contrast, cHCC-CCA and small duct iCCA show strong cytoplasmic K19 expression with a diffuse distribution. 

#### 2.3.3. Immune Microenvironment

A recent study showed that HCC can, in some instances, present with a peculiar immune microenvironment, i.e., with lymphocytic infiltration in the tumor. This was defined as lymphocyte-rich HCC by the 5th WHO classification [7]. Steatohepatitic HCC is also considered to be a type of lymphocytic HCC. The immune microenvironment of HCC is mainly categorized into three groups: immune-high, immune-mid, and immune-low [15]. The immune-high subtype shows marked plasmacytic infiltration as well as infiltration of CD4^+^/CD8^+^ T lymphocytes. This group shows a better prognosis after hepatectomy, even with poor tumor differentiation, and/or stem cell features, such as keratin 19 and/or SALL 4 expression. This data indicates that the immune microenvironment has a greater impact on tumor prognosis than the standard markers of tumor aggression, such as tumor differentiation and stem cell features. Therefore, it is recommended to note the immune cell infiltration of the tumor, especially when it is marked. 

### 2.4. Multiple HCCs: Intrahepatic Metastasis (IH) and Multicentric Occurrence (MC)

**Figure 3:**
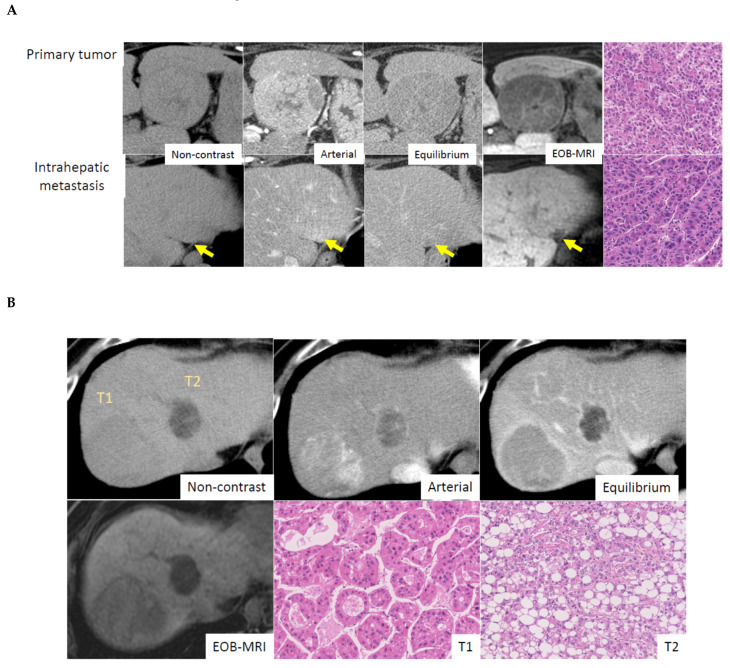
Radiological-, and pathological features in multiple HCC (**A**) Intrahepatic metastasis. Primary tumor shows early enhancement and late washout pattern on CT and shows low intensity on EOB- MRI. Intrahepatic metastatic small lesion shows almost identical enhancement pattern to primary tumor. Histological features are similar between primary and intrahepatic metastatic lesion, which are moderately differentiated HCC with trabecular pattern. Courtesy of Dr. Akihisa UENO (Keio University, Tokyo, Japan). (**B**) Multicentric occurrence. Tumor No.1 (T1) shows early enhancement and late washout pattern on CT and shows uptake of gadolinium ethoxybenzyl diethylenetriaminepentaacetic acid (Gd-EOB-DTPA) on gadoxetic acid-enhanced magnetic resonance imaging (EOB-MRI). Histologically, tumor shows a moderately differentiated HCC with pseudoglandular pattern. Tumor No.2 (T2) shows low attenuation on non-contrast CT, indicating fatty component, and weak early enhancement and late washout. EOB-MRI reveals low intensity, which means no uptake of Gd-EOB-DTPA. Tumor pathology is steatohepatitic HCC. Courtesy of Dr. Akihisa UENO (Keio University, Tokyo, Japan).

Multiple tumors are a frequent phenomenon in HCC, with an incidence around 40–50%. Importantly, multiple HCCs are characterized by two patterns, intrahepatic metastasis (IH) and multicentric occurrence [7]. IH is a multiple tumor composed of a primary tumor associating with multiple smaller tumors, which are metastatic tumors from the primary tumor. The main tumor is usually a moderate and/or poorly differentiated HCC, and the smaller metastatic tumors show similar features to that of the primary tumor. In contrast, multicentric occurrence is considered a de novo cancer, meaning that all the tumors are independent, and their differentiation and morphology are different. The occurrence of MC is significantly higher in HCV-related cases compared with HBV-related cases [16]. Molecular data confirms this, and surprisingly, 30–76% of multiple tumors are found to be MC [17,18]. The distinction between IH and MC can be made by pathological examination. MC cases show HCC containing the portal tracts, considered early HCC, or the presence of early HCC-like areas in the periphery of the HCC, or distinct histology [16]. Figure 3 illustrates the differences between IH and MC.

### 2.5. Histo-Molecular Classification

Histo-molecular classification categorizes HCC into three groups [19]. The first group is a TP53 mutated HCC, which corresponds to G1 to G3. It is considered an aggressive phenotype, characterized by chromosomal instability, cell cycle activation, high AFP levels, and aggressive pathological features. The macro trabecular-massive subtype is frequently seen in this group, especially the G3 subcategory. The second group is the CTNNB1 mutated HCC group, which corresponds to G5 and 6, and is characterized by chromosomal stability and Wnt/b-catenin pathway activation. Pathologically, CTNNB1 mutated HCC frequently shows a cholestatic well-differentiated HCC without inflammation. Beta-catenin mutation is confirmed by IHC. CTNNB1 mutated HCC can be identified by EOB-MRI, which shows a high intensity tumor in the hepatobiliary-phase [20]. The third group is HCC without CTNNB1 or TP53 mutation, corresponding to G4. This group demonstrates a peculiar histological feature, namely steatohepatitic HCC, which presents with steatosis, fibrosis, and inflammation. The IL6/JAK/STAT pathway is activated.

While this classification categorizes HCC and its corresponding histological features, unfortunately the most prevalent oncogenic drivers in HCC, such as TERT, TP53, CTNNB1, AXIN1, are currently ‘not actionable’, meaning they are not targetable by the currently available targeted treatments. A further issue is that the classification is usually made using surgical specimens, which are most likely at early stage HCC. Their molecular landscape might be different from that of advanced HCC, which can be treated by targeted treatment in daily practice.

### 2.6. The Relationship between Pathological Features and Targeted Treatments

#### 2.6.1. Immune Microenvironment and Immune Check Point Inhibitors

An immune-high HCC group with lymphoplasmacytic infiltration was shown to have a high incidence of programed death-ligand 1 (PDL-1) immunoreactivity in tumoral cells in HCCs, as well as infiltrating lymphocytes and macrophages [15]. However, another study showed that PDL-1 immunoreactivity did not correlate with the efficacy of Nivolumab, which is an immune check point inhibitor [21]. This study did not investigate the micro immune environment, such as CD4^+^/CD8^+^ T lymphocyte infiltration, therefore, a correlation between the degree of lymphoplasmacytic infiltration of HCCs and the efficacy of Nivolumab is still unclear. On the other hand, a recent study reported an association between the degree of CD38 immunoreactivity and the response to immunotherapy in HCC [22]. Briefly, a high proportion of CD38 positive cells in HCCs was predictive of a response to immune-checkpoint blockade [22].

#### 2.6.2. CTNNB1 Mutated HCC and Immunotherapy

CTNNB1 mutated HCC does not show any inflammatory cell infiltration, therefore, it is considered to be a ‘cold’ tumor. This indicates the possibility of immunotherapy resistance; immune therapy is more likely to be effective in ‘hot’ tumors, which are defined by the presence of tumor-infiltrating lymphocytes. Indeed, the presence of activating WNT/b-catenin mutations have been found to be associated with innate resistance to immune checkpoint inhibitors [23]. CTNNB1 mutated HCC is able to be recognized by EOB MRI, which shows high intensity tumor in the hepatobiliary phase [20]. As such, performing EOB-MRI is useful in cases where immunotherapy is considered. 

#### 2.6.3. Anti-VEGF Treatment and HCC Pathology

VEGF inhibitors, such as sorafenib, have been used as a first line treatment in advanced HCC for a long time [24]. However, the efficacy is not as good as we would like it to be and there is no biomarker to predict which HCC patients will show a response to the treatment. Fang et al, conducted an elegant study correlating pathological features with sorafenib treatment [25]. They found that vessels that encapsulate tumor clusters (VETC) pattern, which is characterized by the presence of CD34+ vessels completely encapsulating tumor clusters of HCCs, corresponds to rapid tumor dissemination and high recurrence rates. Interestingly, HCC with VETC pattern showed a better response to sorafenib compared to HCC without VETC pattern. This result was also confirmed by the prospective validation cohort. Thus, VETC pattern appears to be a useful predictive histological marker for sorafenib response, and may be used to select patients for sorafenib treatment.

In contrast, Harding et al. performed next generation sequencing on 81 advanced HCC patients who received sorafenib treatment [23]. Tissue samples were taken before treatment, and genomic pathway alterations were correlated with treatment response and outcome. They found that activation of the PI3K–mTOR pathway was associated with poor outcomes in sorafenib-treated patients. Thus, those patients whose HCCs showed activated PI3K-mTOR pathway were NOT good candidates for sorafenib treatment. Since there are some side effects with anti-VEGF treatment, it is useful to be able to predict the efficacy of this treatment.

## 3. Intrahepatic Cholangiocarcinoma (iCCA)

### 3.1. Histological Features: Heterogeneity of Normal Cholangiocytes and iCCA Subtype

**Figure 4:**
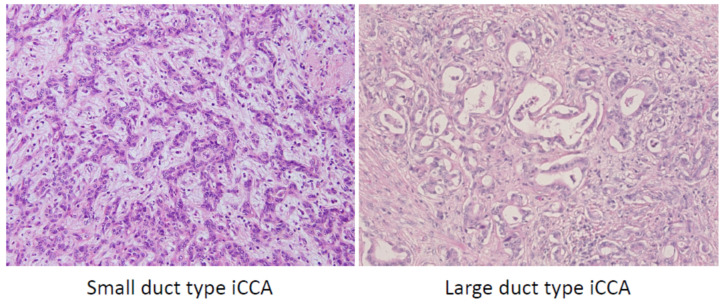
Pathological features of intrahepatic cholangiocarcinoma (iCCA) subtype. Small duct type iCCA shows ductular reaction-like structure with edematous stroma accompanying inflammatory cell infiltration, whereas large duct type iCCA shows clear glandular structure with hyalinized stroma (hematoxylin and eosin staining).

Based on the 5th WHO classification, iCCA is subclassified into two types; small duct type and large duct type [3]. Large duct iCCA occurs in the intrahepatic large bile ducts near the hepatic hilus (proximal to the right and left hepatic ducts), and small duct iCCA mostly arises in the liver periphery. This concept is based on the heterogeneity of normal cholangiocytes, which are lining epithelia of the biliary trees [1]. In brief, small duct comprises cholangiocytes, which are cuboidal shapes without mucin production, while large bile ducts are lined by mucin producing cholangiocytes, which are a cylindrical shape with cilia. These differences are also highlighted by immunohistochemistry and molecular profiles [1,26]. Therefore, it is understandable that iCCA phenotypes differ based on their origin. Large duct type iCCA shows clear glandular structures with mucin production, mimicking normal large bile duct, while small bile duct type iCCA shows cord and/or ductular reaction like tumor structure without mucin production, similar to reactive ductules (Figure 4) [1]. Differences are also observed in their aggressiveness; large duct type iCCA shows very aggressive pathological features, such as perineural invasion and/or lymphatic invasion, and /or lymph node involvement, while small duct type iCCA shows less aggressive features with both lymphatic invasion and perineural invasion being rare, especially in smaller tumor sizes (<3 cm).

Regarding precursor lesions, biliary intraepithelial dysplasia [27], and intraductal papillary neoplasia of the biliary duct [28] are known to be precursor lesions of large duct iCCA, while pre-malignant lesions of small duct iCCA are not clear. Pathological heterogeneity is commonly seen in small-duct type iCCA, while large bile duct iCCA shows rather monotonous pathological features, similar to a Klatskin tumor. This is largely due to the fact that small duct type iCCA originates from small bile ducts, the smallest of which, the canal of Hering, comprises hepatic progenitor cells (HPCs) with the capability of bi-differentiation towards hepatocytes and cholangiocytes.

### 3.2. Macroscopic Features Corresponding to the iCCA Subtype

Intrahepatic CCA presents with four different macroscopic growth patterns; mass forming (MF) type, periductal infiltrating (PI) type, intraductal growing (IG) type, and mixed (MF+PI) type [3]. As indicated by the name, MF type presents as a mass formation in the liver parenchyma. The PI type shows a longitudinal growth pattern along the biliary duct, causing bile duct stenosis. This pattern can also invade the liver parenchyma, resulting in the mixed growth pattern (PI+MF). The IG type presents with an intraductal papillary growth pattern. Note that the IG type is not defined as an iCCA growth pattern by the AJCC/UICC classification [29].

Large duct type iCCA often presents as the PI type, whereas the MF type is typical of small duct type iCCA. As mentioned before, the issue comes with the mixed form (PI+MF), as the predominant growth pattern does not always correspond to the iCCA subtype. Therefore, a tumor biopsy is crucial in order to distinguish between them.

### 3.3. Cholangiolocellular Carcinoma (CLC): Pathological Features and Diagnostic Pitfalls

CLC is a primary biliary cancer characterized as a ductular reaction-like tumor structure (>80% of the tumor). CLC may comprise hepatocytic differentiation in the periphery of the tumor, and/or a large duct type iCCA component in the center of tumor. Due to its histological diversity, CLC is thought to originate from HPCs [30]. CLC used to be categorized as cHCC-CCA in the previous WHO classification, however, the 5th WHO classification categorized CLC into small duct iCCA or cHCC-CCA, depending on the hepatocytic component [3]. In brief, if CLC comprises hepatocytic differentiation, it is categorized to be cHCC-CCA. If not, it is considered to be small duct type iCCA. 

There are some issues with this new classification. Firstly, the classification of CLC depends on the presence of an hepatocytic component. However, the definition of hepatocytic differentiation is not clearly defined, especially the type of immunohistochemistry used. Currently, several different immunohistochemistry screens are used to identify hepatocytic differentiation, but their sensitivity and specificity are not all the same [2]. This issue needs to be clarified as soon as possible, otherwise the clinical outcome and molecular profiles will be biased if tumor categorization is not done in a similar manner. A further problem is that identification of hepatocytic component could be underestimated, especially in biopsy samples. Therefore, combining this data with imaging will be essential to understand the tumor characteristics if there is any discrepancy between clinical interpretation and pathological aspects.

### 3.4. Intrahepatic CCA Subtype and Treatment Options

Characterizing the iCCA subtype is crucial as it affects treatment choice. This is because IDH1/2 mutations and FGFR2 fusion are iCCA specific changes that are observed in 10–30% of small duct iCCAs, and treatable with currently available treatments. In brief, ivosidenib, an oral inhibitor of the mutated IDH1 enzyme, shows efficacy in iCCA patients in terms of median progression-free survival date, and median overall survival rate [4]. When looking at the FGFR2 fusion, several FGFR inhibitors show a good response in iCCA patients [5,6].

Approximately 70% of iCCA patients are diagnosed at an advanced and inoperable stage and having a promising treatment option would be great news for these patients, as opposed to the palliative treatment they currently receive. Therefore, once again, achieving an accurate diagnosis is crucial to provide the best possible treatment for the patients.

### 3.5. Differential Diagnosis: Large Duct iCCA and Small Duct iCCA

**Figure 5:**
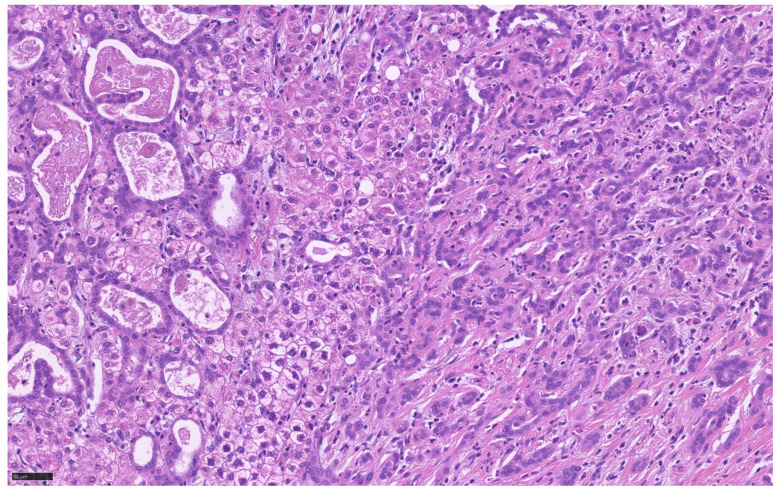
Tumor heterogeneity of cHCC-CCA which exhibits large duct iCCA component (left), HCC component (middle), and small duct iCCA component (right).

The differential diagnosis that you need to keep in mind is different between large duct iCCA and small duct iCCA. In the case of large duct iCCA, the most important differential diagnosis is a metastatic tumor, particularly of pancreatic origin [31]. This is because the pathological features are quite similar in both tumors. Immunohistochemistry, such as SMAD4, MUC5AC, and CDX2 expression, may be useful to distinguish them; SMAD4 nuclear expression is more preserved in iCCA compared with pancreatic cancer. In contrast, CDX2 and MUC5AC expression are more common in pancreatic cancer compared with iCCA. [31]. However, it is not always perfect or reliable, therefore, a comprehensive diagnosis that includes clinical information and imaging, is essential to make a definitive diagnosis. In the case of small duct type iCCA, it needs to be distinguished from cHCC-CCA because cHCC-CCA may contain small duct iCCA features as part of its tumor heterogeneity (Figure 5). Identification of hepatocytic differentiation is the key to distinguish cHCC-CCA and small duct iCCA. This would be easy to do in a surgical specimen as whole tumor aspects are able to be assessed, but may be very challenging using a tumor biopsy. Firstly, the proportion of hepatocytic differentiation can be limited in cHCC-CCA and secondly, the pathological diagnosis can only be made on a limited sample, which does not always reflect all tumor features of the tumor. Therefore, a needle biopsy has the risk of underestimating the hepatocytic differentiated area. In this case, a combination of clinical assessment and imaging would be helpful to understand the overall view of the tumor. It would be acceptable to perform a molecular test to assess whether the tumor harbors the IDH1/2 mutations or FGFR2 fusions, especially in inoperable cases, as this information will open the possibility for targeted treatment.

### 3.6. Molecular Landscape of iCCA

Several studies report that iCCA and extrahepatic CCA have a different molecular profile [32]. Targetable molecular changes, such as IDH and FGFR, are more frequently seen in iCCA. Etiology also corresponds to the molecular subgroup. For instance, fluke-positive iCCA (associated with a liver fluke infection, such as *Opisthorchis viverrini* and *Clonorchis sinensis*) presents with TP53, ARID1A, and ERBB2 mutations, whereas fluke-negative iCCA (not associated with a liver fluke infection) harbors the FGFR, BAP1, and IDH1/2 mutations [33]. The fluke-positive group have a poorer prognosis compared with the fluke-negative iCCA group [33]. In terms of growth pattern, only one study has described that the IDH1/2 mutation is only seen in MF type iCCA and CLC [34,35]. Thus far, there are no molecular studies based on the small and large duct iCCA categories. Etiology and growth pattern most likely correspond to iCCA subtype (large and small duct type), however, it is essential to perform molecular studies based on small duct and large duct iCCA classification to achieve the precise molecular landscape of iCCA.

## 4. Combined HCC-CCA

### 4.1. Histological Features

Combined HCC-CCA is a tumor comprising both hepatocytic and cholangiocytic differentiation. Both areas show transitional features, different from a collision tumor that contains separate HCC and iCCA and no transitional features. The definition of cHCC-CCA by the 5th WHO classification corresponds to type 3 tumor, as described by Allen and Lisa [36], and type II (transitional neoplasms), as defined by Goodman and colleagues [37]. The percentage of each component and their tumor differentiation grades vary. As such, histological aspects may vary in each case, leading to diagnostic challenges. In addition, so-called typical cHCC-CCA, presenting as clear HCC and iCCA components with transitional features, accounts for around 17% of all cases [38]. The rest show a mix of varying tumor structures and tumor differentiation. Therefore, immunohistochemistry is necessary to confirm their features.

### 4.2. Diagnostic Pitfalls and Utility of Radiological Images

Although the pathological features of cHCC-CCA are complex, diagnosis is less problematic with surgical specimens, as the whole tumor features can be assessed. An accurate diagnosis using a tumor biopsy can, however, be challenging. This is largely due to the fact that a biopsy based diagnosis is based only on what is seen in the biopsy and must comprise both hepatocytic and cholangiocytic features in order to diagnose cHCC-CCA. In reality, however, this is not easy. As such, combining biopsy results with imaging is essential for a cHCC-CCA diagnosis. Likewise, images are influenced by the histological heterogeneity, especially the proportion of each component in cHCC-CCA. For instance, cHCC-CCA, with a predominant HCC component, resembles images of HCC [39,40], while cHCC-CCA, with a predominant iCCA component, mimics small-duct type iCCA [39]. The area for the biopsy can be determined by the imaging findings, and, if necessary, two biopsies from different imaging patterns may be helpful to achieve diagnostic accuracy. We have to keep in mind that a biopsy diagnosis alone may be at risk of under diagnosis of cHCC-CCA.

### 4.3. Differential Diagnosis

As described before, cHCC-CCA needs to be differentiated from small duct iCCA, K19 positive HCC, and CLC. For more detail, please refer to each diagnostic section.

### 4.4. Pathological Features and Treatment

There is no global consensus for the optimal therapy for unresectable/metastatic cHCC-CCA patients. Kim et al., report no significant difference regarding efficacy outcomes between patients treated with sorafenib, which is usually used for HCC, and cytotoxic chemotherapy, which is usually used for iCCA. Unfortunately, the pathological features, such as predominant histological type, were not helpful for determining the therapeutic agent, or assessing patient outcome [41].

While cHCC-CCA is indeed composed of hepatocytic and cholangiocytic components, its carcinogenesis is totally different from HCC or iCCA, as cHCC-CCA does not originate from either hepatocytes or cholangiocytes. Therefore, the current available treatments for HCC or iCCA may be of no use with cHCC-CCA. A molecular profile will be important to find promising treatment options.

### 4.5. Molecular Profiles

The molecular profile of cHCC-CCA varies, as does the pathological features. There are several interesting studies regarding molecular profiles [42,43,44,45,46], but the molecular data varies depending on the study. For instance, IDH mutations, which are often seen in small duct type iCCA, were found in some studies [42,45,46] but not in others [43,44]. A possible explanation could be the different predominant features of the sample tissues and/or different diagnostic criteria. Since cHCC-CCA is a rare aggressive tumor with no current standardized therapy option in unresectable/metastatic cHCC-CCA patients, we need to investigate the molecular profile to identify actionable genes that can be therapeutically targeted. In addition, using well-characterized and classified tumor tissue is essential to best characterize the molecular profile. 

## 5. Importance of PLC Diagnosis for Prognosis and Treatment Options

As has been described in the preceding sections, characterization of each PLC is necessary. From a broad perspective, however, discrimination of each PLC tumor type is vital as the prognosis and treatment choice differs.

With respect to treatment choice, only HCC is currently eligible for liver transplantation (LTx). This is mainly due to the fact that HCC does not normally show any lymphatic invasion or lymph node involvement, as in the case of iCCA and/or cHCC-CCA. Therefore, distinguishing between HCC and non-HCC is essential, especially when LTx is considered as a treatment option. The imaging pattern of small duct type iCCA and cHCC-CCA could be similar to that of HCC, and a tumor biopsy is recommended if there is any doubt. In this setting, there are two things that need our attention. Firstly, the pathologist should discriminate HCC from cHCC-CCA. If the tumor sample contains ductular area, regardless of its proportion, it is important to perform immunohistochemistry to determine whether it is a cholangiocytic differentiation or not. If there is any doubt, it is strongly recommended to send the tissue sample to an expert liver pathologist for a second opinion. This is also recommended by the EASL guidelines [47]. Another important point is the area of tumor biopsy. In this setting, the area of tumor biopsy, especially in the case of cHCC-CCA, is critical, as distinguishing HCC from cHCC-CCA is challenging [48]. It is important to take a sample from the non-typical HCC imaging area as it corresponds to cholangiocytic differentiated area.

With regard to prognosis, the overall survival rate is significantly better in HCC compared to iCCA and cHCC-CCA. When comparing iCCA and cHCC-CCA, cHCC-CCA tends to show better outcome compared to iCCA, but without statistical value [48,49]. Table 1 summarizes the histological subtypes of PLCs and their prognosis. This table shows the histological parameters of each tumor type corresponding to better or worse prognosis, as compared with the prognosis of each category and tumor type. These data indicate that the distinction of tumor type, as well as tumor subtyping, are very important despite the difficulty in doing so.

## 6. Future Perspectives: The Role of Pathologists in PLC Diagnosis

A pathological diagnosis of primary liver cancer becomes crucial, not only in the initial diagnosis, but also for subtyping of the tumor. This information is critical for predicting tumor aggressiveness and treatment choice, and makes the role of the pathologist essential in these cases.

In the case of HCC, tumor aggressiveness corresponds to the specific subtype, such as macrotrabecular-massive type, K19 immunoreactivity [13], CTNNB1 mutation, and micro immune environment [15]. In addition, characterization of multiple HCCs is critical to improve patient outcome especially in those patients scheduled for systematic treatment. This is because the efficacy of treatment will vary in MC cases due to the individual nature of all the component tumors. A pathological interpretation is useful to distinguish between IH and MC.

When it comes to iCCA, identifying the subtype is essential for treatment choice. It is also important to differentiate large duct iCCA from metastatic tumor, and differentiate small duct iCCA from cHCC-CCA. If the pathological features are discrepant with clinical features, a comprehensive approach is strongly recommended to achieve a definitive diagnosis.

With regards to cHCC-CCA, identification of hepatocytic differentiation is the key for diagnosis. Therefore, it is important to establish the definition of hepatocytic differentiation in order to diagnose cHCC-CCA in a standardized manner.

Last but not least, it is essential to establish precise molecular profiling, as well as morpho-molecular classification, of iCCA based on its subtype, as well as cHCC-CCA. Identifying actionable mutations are key to improving patient outcome in those aggressive tumors. In addition, establishing morpho-molecular classification in iCCA and cHCCA-CCA would be helpful for the tumor diagnosis, especially when using tumor biopsy. 

To achieve this, it is critical that all tumor samples be diagnosed in the same manner; without proper tumor characterization and categorization, it is not possible to establish a trusted/reliable mutation landscape. In addition, it is important that tumor classification be made based on the latest WHO classification. Since the tumor categorization has changed between the 4th and 5th WHO classification, caution is needed when assessing any publication using the 4th WHO classification. 

## 7. Conclusions

PLCs are heterogenous tumor populations. Understanding the key features of this heterogeneity is crucial in terms of how it affects patient outcome and treatment choice. Tumor biopsies are helpful to achieve this but in some cases a multi-pronged approach utilizing biopsy, clinical assessment, and imaging may be required for an accurate diagnosis and therapeutic strategy. In addition, molecular profiling will certainly assist the process of accurate diagnoses and targeted therapy. Therefore, multicenter collaborative works are recommended to establish a standardized morpho-molecular classification in iCCA and cHCC-CCA.

## Figures and Tables

**Table 1:** Histological subtypes of Primary liver cancers (PLCs) and prognosis.

	Prognosis	
Hepatocellular Carcinoma (HCC)	Better	Worse
Histological subtypes	Lymphocyte-rich Clear cell	Macrotrabecular-massive Neutrophil-rich
Tumor differentiation	well	poor
Immunohistochemical subtype	K19 negative HCC	K19-positive HCC
Immuno microenvironment	Immune-high	Immune-low
**Combined HCC-CCA**		
Tumor component	Predominant HCC component	Predominant iCCA component
**Intrahepatic cholangiocarcinoma (iCCA)**		
Histological subtypes	Small duct type	Large duct type

As compared with the prognosis of each category.

## Data Availability

Not applicable.
